# Cancer in an Historic Washington DC African American Population and Its Geospatial Distribution

**DOI:** 10.3389/fonc.2018.00383

**Published:** 2018-11-13

**Authors:** Latifa Jackson, Hasan Jackson, Mariam Mohammed, Nicholas Guthrie, Shihyun Kim, Rita Okolo, Fatimah Jackson

**Affiliations:** ^1^W. Montague Cobb Research Laboratory, Howard University, Washington, DC, United States; ^2^Department of Pediatrics, College of Medicine, Howard University, Washington, DC, United States; ^3^Global Research & Interdisciplinary Development, Washington, DC, United States; ^4^Jackson Wellness Group, Bethesda, MD, United States; ^5^College of Medicine, Howard University, Washington, DC, United States; ^6^Department of Biology, Howard University, Washington, DC, United States

**Keywords:** Cobb Collection, historic cancer, health disparities, population health, mid-Atlantic region, black Americans

## Abstract

**Background:** Cancer continues to be a major cause of morbidity and mortality in the African American community but insights into the types and incidence of cancer 85 years ago have been virtually non-existent and little is known of its geospatial distribution. Historical information on cancer can shed light on current health disparities, particularly among African Americans.

**Objective:** The aims of this study were to: (1) assess the frequencies of the cancer types present among Cobb Collection individuals; (2) compare these data with current research on cancer in African Americans; and (3) evaluate the pattern of cancer expression, including its geospatial distributions, as a cause of death between 1931 and 1969 in an historic African American subgroup and compare this pattern with the historic and contemporary patterns of cancer etiology and incidence.

**Methods:** Systematic assessments of the existing clinical, demographic, and anatomical records in the Cobb Research Laboratory were made of individuals identified as dying from specific cancers from 1931 to 1969. These were compared with the national profiles of cancer during the historic time an individual died as well as the contemporary patterns of cancer deaths. Analysis of their residential addresses just prior to death were assessed using a commercial geographic information system. Each location was assigned a geospatial location and proximity between each site and clusters of sites were investigated.

**Results:** Seventeen different cancer types were found within 28 individuals of the Cobb Collection between 1931 and 1969. The cancer types with the highest frequencies were carcinoma of stomach, lung, esophagus, larynx and bronchogenic carcinoma. Eighty-four percent of all cancer incidents occurred in males and 76% were among individuals identified as African American. Seventy-one percent of the highest incidence cancers were among African American males. Geospatial clustering was observed most noticeably in the redistribution of carcinoma of the esophagus.

**Conclusion:** Our results provide historical depth to our knowledge of the common cancer causes of morbidity among African Americans of Washington DC from 1931 to 1969. We contrast these findings with national historical data on cancer etiology and ethnic disparities in incidence. Our study suggests that historic data can provide longitudinal depth to our understanding of the persistence of cancer susceptibilities in a vulnerable subgroup.

## Introduction

African Americans (AA) account for 12% of the population in the United States yet have the highest death rate and shortest survival for most cancers than any other ethnic group in the country. The American Cancer Society estimated 189,910 new cancer diagnoses in the AA community in 2016. In AA males, the two most common diagnosed cancers are prostate (31% of all cancers) and lung (15%) while in AA females breast (32%) and lung (11%) predominate. The 2016–2018 *Cancer Facts and Figures for African Americans*, released by the American Cancer Society shows that the incidence for all cancers had increased from the mid-1970s to the early 1990s (1–4). However, what was the epidemiology of cancer prior to the 1970's and how was it geospatially distributed? At the W. Montague Cobb Research Laboratory at Howard University, the Cobb Skeletal Collection of human skeletal and dental remains contains a wealth of information in clinical records and anatomical materials that can illuminate the history of cancer in the AA community over a 37-year span, between 1931 and 1969 (5–7).

In this study, we investigate cancer in the Cobb Collection to specifically characterize the various cancer types that caused mortality within the collection, analyze trends found to occur between 1931 and 1969, and evaluate a link between these historic findings on cancer epidemiology and etiology and the current statistics on cancer rates within the AA community. We also include data on the geospatial distribution of cancer in this cohort to look for evidence of temporal or spatial clustering of specific cancers. The motivation for this research is the unprecedented rise in cancer rates in the AA community (8), as well as the higher incidence rates (3) and aggressiveness of certain cancer types in AA, compared to groups with other ancestral and environmental backgrounds (9). Our historical consideration of cancer may shed light on the current cancer disparities affecting this vulnerable population.

The W. Montague Cobb Research Laboratory, founded in 1931 by Dr. William Montague Cobb, houses two major collections of archeological remains with over 400 years of biological significance to the nation and particularly to its AA community (5). The oldest of these collections is the New York African Burial Ground remains from the seventeenth to eighteenth centuries. The second collection and the focus of this investigation is the Cobb Collection. The Cobb Collection contains the remains of 699 individuals from the nineteenth to twentieth century with 83% of the collection representing AAs from the Washington, District of Columbia area (7).

Among AAs historically, detailed medical insights of past generations are not readily available or adequately studied (10–13). The protection of precious bioarcheological remains and the history of African descendent populations and their diasporas within the United States is amiss; no legal backing similar to the Native American Grave Protection and Repatriation Act (NAGPRA) is currently available to ensure and guarantee the safe-handling, respectful care and transition of power to descendants or the AA community (14). No legislation exists to ensure that the systematic research on AA skeletal materials is undertaken to fill in the gaps in our understanding of the historical depths of the health disparities currently afflicting AAs. The sciences of biological anthropology and archeology hold many promises of the discovery of links and lineages to the past that can help us better understand the present medical demographics and aid the design of future precision medical science (15). The Cobb Collection provides an avenue to enrich the study of AA history scientifically, protect the legacy of past ancestry through the curation of the remains, and provide the necessary linkage between the past medical struggles within the AA community and the current health disparities and its geospatial and environmental components. This may then allow researchers to find reasonable and appropriate solutions for the future improvement in health.

## Materials and methods

Using our IRB-exemption for studying historic skeletal and dental remains, CITI-certified researchers evaluated each of the clinical records of the Cobb Collection to identify the individuals who had died of various cancers between 1931 and 1969. Using visual inspection of the data, raw data was sequestered from the Cobb Research Laboratory's Cobb Collection database using keywords such as “cancer,” “carcinoma,” “tumor,” “outgrowths,” and “neoplasia.” Once identified, these individuals were cross-checked and the specific cancer types noted, the frequency of occurrence for the cancer types was documented, and the last residential address of the affected individual noted. Demographic information on each individual dying from cancer was recorded, including name, age, and gender, and investigated using existing Census Bureau and other public databases to reconstruct individual biohistories. Autopsy records of those individuals whose deaths were attributed to cancer as either a primary or secondary cause of death were set aside for further study.

Within the Cobb Collection's clinical records, multiple types of cancers were evident. These cancers were recorded in terms of five factors: epidemiology, etiology, symptomatology, diagnosis, and treatment as reported at the original time of diagnosis. As diagnostic criteria have varied over the decades of awareness of cancer as a cause of morbidity and mortality, the temporal assessment of specific cancers was reviewed within the context of the knowledge of the time. Archival records on the definitions and interpretations of the specific cancers observed in the Cobb Collection at the time of diagnosis were sought and we were successful in locating the ICDs for the relevant periods (16). Additionally, we collected historical data on cancer types from the published literature.

Our working hypothesis was that the historical record of cancer evident in the Cobb Collection that is due to ancestral genetics parallels the current health disparities in cancer incidence among contemporary African Americans yet the cancer-relevant environment has changed over the decades. Therefore, the historical record can best serve as a limited predictive index of long term susceptibility in this population. To enhance the applicability of our historical data, current, published epidemiological trends in specific cancers were studied for comparisons with our historical data.

**Figure 1:**
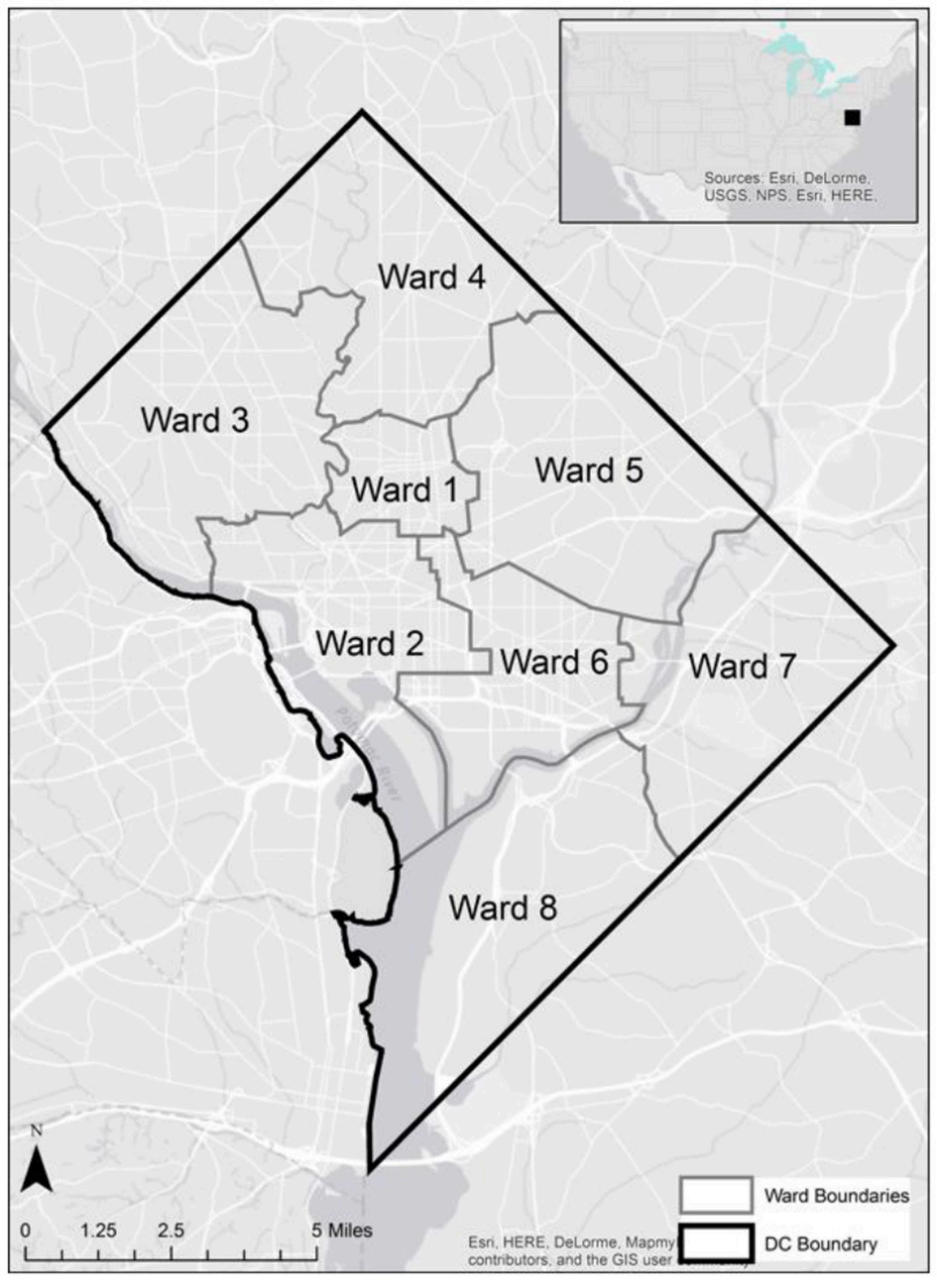
Contemporary incidence of cancer (morbidity) by US ethnicity and gender 1975–2013. Data from the National Cancer Institute shows that, while the overall incidence rate of all cancers has increased from 1990 to 2000, the overall mortality rate of all cancers has decreased over time (25).

Geospatial data on the residence of cancer victims just prior to the deaths were identified from the original demographic data and plotted digitally on historical and contemporary maps of the greater Washington DC Metropolitan region. Ward boundaries were used to delineate regional residential areas. Addresses of individuals from the Cobb Collection were geocoded and imported into a geographic information system as point features. Each address was assigned a geospatial location in latitude and longitude. These data were then overlaid onto the ward boundaries to examine spatial relationships between addresses and ward boundaries (see Figure 1).

Spatiotemporal analysis of the Cobb collection addresses was examined through the grouping of cancer deaths by decade. Each decade (i.e., 1930s, 1940s, 1950s, 1960s) were filtered and examined separately to assess spatial patterns within each decade time period and persisting patterns between decades.

In addition, the mortality related-cancer associated with each address was examined. The specific cancer type was overlaid on the ward boundaries. The diversity of cancer type in space was described and presented. Cancer types were also classified and mapped based upon organ groupings (e.g., digestive, neurological, and reproductive) to determine the presence of spatial clusters.

## Results

In the Cobb Collection, there are a total of 987 individuals, 780 of which have records of cause of death. From the records that are present, there are 26 individuals whose primary cause of death was cancer of some type. There are two individuals with cancer as a secondary cause of death. Of these 28 individuals, 24 were male and 4 were female. Demographic information revealed 14 individuals were classified as Negro, 7 were classified as Colored, and 7 were identified as White. For the purposes of this study, Negro and Colored are collapsed into a single category and designated as African American (AA). White individuals in the collection are reclassified as European American (EA).

### Cancer types in the cobb collection

Seventeen different cancer types were documented (Table 1) among the individuals of the Cobb Collection. The average age of death of these individuals was 65.5 years. The total number of men dying of cancer was 24 (mean age 58.8 years) while the number of women in the Cobb Collection dying of cancer was 4 (mean age 53.8 years). Cancers of the brain, pancreas, skin, and ovary were missing for years 1931–1957 from the Cobb Collection.

**Table 1:** Specific kinds of cancer observed in the Cobb Collection.

**Cancer type**	**Frequency 1931–1969**	**Age range**	**Gender ratio Male:Female**	**Year of death**
1. Carcinoma of uterus	1	66	0:1	1931
2. Carcinoma of stomach	2	51–69	2:0	1934, 1952
3. Carcinoma of kidney	1	62	1:0	1943
4. Bronchogenic carcinoma	4	50–78	4:0	1944, 1948, 1953, 1959
5. Carcinoma of lung	3	57–62	3:0	1946, 1955, 1958
6. Carcinoma of esophagus	4	39–65	3:1	1949, 1952, 1952, 1958
7. Bowel obstruction carcinomatosis	1	60	0:1	1956
8. Carcinoma of the penis	1	54	1:0	1957
9. Carcinoma of the prostate	1	72	1:0	1957
10. Carcinoma of larynx	2	63–65	2:0	1957, 1958
11. Carcinoma in neck	1	58	1:0	1958
12. Carcinoma of colon	1	67	1:0	1959
13. Cancer of cervix	1	50	0:1	1955
14. Cancer of tongue	1	65	1:0	1944
15. Carcinomatosis	2	49–65	2:0	1952, 1953
16. Cancer of plasma cells	1	49	1:0	1952
17. Neurofibrosarcoma	1	22	1:0	1959

### Body regions exhibiting cancer in the cobb collection

In the early years of cancer diagnosis in the United States, identification of cancer was largely limited to specific regions of the body exhibiting abnormal cell growth. In Table 2 the variation in the areas of the body affected by cancer among the Cobb Collection individuals is displayed. Clear evidence of increased vulnerability to death by cancers affecting the respiratory system is observed among the men of the Cobb Collection. A lesser but important disparity is also observed among cancers of the digestive system with more males affected than females. It is not known whether the cancers Dr. Cobb diagnosed were localized to the site of the origin when initial post-mortem diagnosis was done. Some of these may have been metastasized and could have been detected away from the site of origin. Breast cancer started to appear in the Cobb Collection between 1958 and 1969. Its presence could be attributed to changes in lifestyle or other environmental factors.

**Table 2:** Cancer types in the Cobb Collection by primarily affected specific body areas.

**Cancer type**	**Number of affected individuals (1931–1969)**	**Gender ratio Male:Female**
Respiratory cancers	10	10:0
Digestive cancers	9	7:2
Urogenital cancers	2	2:0
Reproductive cancers	3	1:2
Neurological cancers	1	1:0
Hematopoietic and lymphatic system cancer	1	1:0
Other	2	2:0

### Clinical details of cancer in the cobb collection

In Table 3 the current definitions and clinical details of the 17 cancer types observed in the Cobb Collection are presented. These definitions are from the National Cancer Institute (www.cancer.gov)^1^ and reflect contemporary definitions of these cancers.

**Table 3:** Clinical details of the cancers reported in the Cobb Collection.

**Type of cancer reported in the cobb collection**	**Elaborated definition of specific cancer**
Carcinoma of uterus	There are two types of uterine cancer, based on what tissue the malignancy begins. Most uterine cancers begin in the endometrium and are classified as endometrial cancer, most commonly beginning in cells that make mucous (adenocarcinomas). Less common, is uterine sarcoma that begins in cells that contribute to muscle tissue that support the structure of the uterus. Endometrial cancer is more likely to be cured than uterine sarcoma.
Carcinoma of stomach	Stomach cancers, also called gastric cancer, occurs in cells in the lining of the stomach. Most gastric cancers begin in cells that make and release fluids, called adenocarcinomas. Less common are gastrointestinal carcinoid tumors, stromal tumors, and lymphomas. The most common cause of gastric cancer is infection by the bacteria *H. pylori*.
Carcinoma of kidney	Kidney cancer, also called renal cancer, has three main types. The most common type begins in the cells that make urine in the renal pelvis; renal cell in adults and Wilms tumor in children.
Bronchogenic carcinoma/carcinoma of the lung-	Lung cancers are divided by two main groups; non-small cell, which is the most common, and small cell. Lung cancer is the leading cause of cancer death in America.
Carcinoma of esophagus	Esophageal cancers are often diagnosed in late stage secondary to no early signs or symptoms. This type of cancer can start in the cells lining the walls of the esophagus, squamous cell, or in the cells that make and release fluids, adenocarcinoma.
Bowel obstruction carcinomatosis	Malignant bowel obstruction arises as a complication from abdominal, ovarian, or colon cancers. It most often involves the small intestine, but obstruction in the large intestine is not uncommon. In advanced ovarian cancer, death from malignant bowel obstruction is as high as 42% in the United States (17).
Carcinoma of the penis	Penile cancer most often forms in the squamous cells on or underneath the foreskin. This type of cancer is curable if detected early. A third of all diagnosed penile cancer is caused by infection of the human papillomavirus (HPV).
Carcinoma of the prostate	Prostate cancer is the second most common cause of cancer deaths in males in the United States. Most prostate cancers are adenocarcinomas, and there are no early signs or symptoms, which makes this cancer often diagnosed in advanced stage if not screened before the onset of symptoms. Weak urine flow or frequent urination are not unique to prostate cancer, and could be from benign conditions.
Carcinoma of larynx/Carcinoma in neck/Cancer of tongue	Laryngeal cancers, classified by its involvement of the head and neck, begin in squamous cells that line the throat's voice box. Mouth, lips, salivary gland, nose, and neck cancers have a similar pathology.
Carcinoma of colon	Most colorectal cancers as adenocarcinomas that begin as polyps, and can develop into malignancies over time. Screening via colonoscopies have improved early detection and prevention of cancerous polyps
Carcinomatosis	General term for cancer, unspecified
Cancer of cervix	Cervical cancer affects the lower, narrow end of the uterus. Like uterine cancer, cervical cancer can present as squamous cell or adenocarcinoma.
Cancer of plasma cell	Plasma cell carcinoma affects the cells in the immune system that are responsible for making antibodies. The malignancy creates overproduction of abnormal plasma cells in the bone marrow, which then may cause thickening of the blood, renal disease, bone, and soft tissue tumor growth. If an individual has a single tumor, the cancer is classified as plasmacytoma. If there are multiple tumors, it is classified as multiple myeloma, which is often diagnosed in advanced stage due to no early symptoms. Plasma cell cancer cause a slew of other diseases due to the nature of its proliferation in the body.
Neurofibrosarcoma-	Neurofibrosarcoma is classified by malignant peripheral nerve sheath tumors. Benign tumors have a slow onset, while malignant tumors of the nerve sheath develop rapidly and cause pain, neurological deficit, damage to surrounding tissue, and mass effect.

### Comparison of the cancer types observed in the cobb collection with historic cancer categories in published reports of the ICD (ICD) for oncology

When Dr. W. Montague Cobb began his evaluations of cadavers donated to the Cobb Collection, he would have used the 1929 ICD-4 classifications of cancer. As the donations continues to build the Cobb Collection, Dr. Cobb would have used the latest ICD reports to diagnose cancer as a cause of death. In Tables 4–7 we present a comparison of the historic cancers observed in the Cobb Collection individuals with the cancer categories that were in place at the time of initial post-mortem diagnosis. In Table 8 we collapse these specific cancer types by primary affected body region and contrast these by the most current ICD report publication date. It is important to note that not all cancer categories that were recognized in these published reports are present in the individuals of the Cobb Collection. This discrepancy may reflect a variance in susceptibility of the Cobb Collection individuals from the national norms, gender imbalance among the individuals of the Cobb Collection, the average older age of the individuals of the Cobb Collection, or other unknown factors.

**Table 4:** Comparison of published ICD-4 (1929) Cancer categories with types reported in Cobb Collection 1931–1937.

**ICD number-4(1929)**	**ICD class of cancer published in 1929**	**Cancer type reported in Cobb Collection in 1931–1937**
45	Cancer of the buccal cavity and pharynx	Not observed
46	Cancer of the digestive organs and peritoneum	Cancer of the digestive organs and peritoneum observed
47	Cancer of the respiratory organs	Not observed
48	Cancer of the uterus	Cancer of the uterus observed
49	Cancer of the other female genital organs	Not observed
50	Cancer of the breast	Not observed
51	Cancer of the male genitourinary organs	Not observed
52	Cancer of the skin	Not observed
53	Cancer of the other or unspecified organs	Not observed
54a	Non-malignant tumors of the female genital organs	Not observed
54b	Non-malignant tumors of the other sites	Not observed
55a	Tumors of undetermined nature of the female genital organs	Not observed
55b	Tumors of undetermined nature of the other sites	Not observed

**Table 5:** Comparison of published ICD-5 (1938) Cancer categories with types reported in Cobb Collection 1938–1947.

**ICD number-5(1938)**	**ICD class of cancer published in 1938**	**Cancer type reported in Cobb Collection in 1938–1947**
45a	Cancer of the lips	Not observed
45b	Cancer of the tongue	Cancer of the tongue
45c	Cancer of other or unspecified parts of buccal cavity and pharynx	Not observed
46a	Cancer of the esophagus	Cancer of the esophagus
46b	Cancer of the stomach and the duodenum	Not observed
46c	Cancer of the intestines other than duodenum or rectum	Not observed
46d	Cancer of the rectum	Not observed
46e	Cancer of the liver and biliary passages	Not observed
46f	Cancer of the pancreas	Not observed
46g	Cancer of the peritoneum	Not observed
46h	Cancer of other or unspecified digestive organs	Not observed
47a	Cancer of the larynx and trachea	Cancer of the larynx and trachea
47b	Cancer of the lung and pleura	Cancer of the lung and pleura
47c	Cancer of unspecified respiratory organs	Not observed
48a	Cancer of the uterus specified as cancer of cervix	Not observed
48b	Other or unspecified cancer of the uterus	Not observed
49	Cancer of other female genital organs	Not observed
50	Cancer of the breast	Not observed
51a	Cancer of the scrotum	Not observed
51b	Cancer of the prostate	Not observed
51c	Cancer of other or unspecified male genital organs	Not observed
52	Cancer of the urinary organs	Cancer of the urinary organs
53	Cancer of the skin (scrotum excepted)	Not observed
54a	Glioma (not specified as benign)	Not observed
54b	Sarcoma	Not observed
54c	Other or unspecified forms of cancer of the brain and other parts of the nervous system	Not observed
55a	Cancer of the adrenal glands	Not observed
55b	Cancer of the bones	Not observed
55c	Cancer of the thyroid gland	Not observed
55d1	Cancer of the nose or nasal cavity	Not observed
55d2	Cancer of other unspecified organs	Not observed
56	Non-malignant tumors…	Not observed
56a	Of the ovaries	Not observed
56b	Of the uterus	Not observed
56c	Of other female genital organs	Not observed
56d	Of the brain and other parts of the nervous system	Not observed
56e	Of other and unspecified organs	Not observed
57	Tumors of undetermined nature…	Not observed
57a	Of the ovaries	Not observed
57b	Of the uterus	Not observed
57c	Of other female genital organs	Not observed

**Table 6:** Comparison of ICD-6 (1948) Cancer categories with types reported in Cobb Collection 1948–1957.

**ICD number-6(1948)**	**ICD class of cancer published in 1948**	**Cancer type reported in Cobb Collection in 1948–1957**
140–148	Malignant neoplasm of buccal cavity and pharynx	Malignant neoplasm of buccal cavity and pharynx
150–159	Malignant neoplasm of the digestive organs and peritoneum	Malignant neoplasm of the digestive organs and peritoneum
160–165	Malignant neoplasm of the respiratory system	Malignant neoplasm of the respiratory system
170–181	Malignant neoplasm of the breast and genitourinary organs	Not observed
190–199	Malignant neoplasm of other unspecified sites	Not observed
200–205	Neoplasms of lymphatic and hematopoietic tissues	Neoplasms of lymphatic and hematopoietic tissues
210–229	Benign neoplasm	Not observed
230–239	Neoplasm of unspecified nature	Not observed

**Table 7:** Comparison of ICD-4 (1958) Cancer categories with types reported in Cobb Collection 1958–1969.

**ICD number-7(1958)**	**ICD class of cancer published in 1958**	**Cancer type reported in Cobb Collection in 1958–1969**
140–148	Malignant neoplasm of buccal cavity and pharynx	Not observed
150–159	Malignant neoplasm of the digestive organs and peritoneum	Malignant neoplasm of the digestive organs and peritoneum
160–165	Malignant neoplasm of the respiratory system	Malignant neoplasm of the respiratory system
170–181	Malignant neoplasm of the breast and genitourinary organs	Malignant neoplasm of the breast and genitourinary organs
190–199	Malignant neoplasm of other unspecified sites	Malignant neoplasm of other unspecified sites
200–207	Neoplasms of lymphatic and hematopoietic tissues	Not observed
210–229	Benign neoplasm	Benign neoplasm
230–239	Neoplasm of unspecified nature	Not observed

During the historic periods of the Cobb Collection, cancer increased in incidence as a cause of death and shifted from primarily digestive cancers (during ICD6) to more respiratory cancers (in ICD7). Overall, respiratory and digestive cancers account for 65% of all cancers from 1931 to 1969.

### Epidemiological trends in cancer in the cobb collection

**Figure 2:**
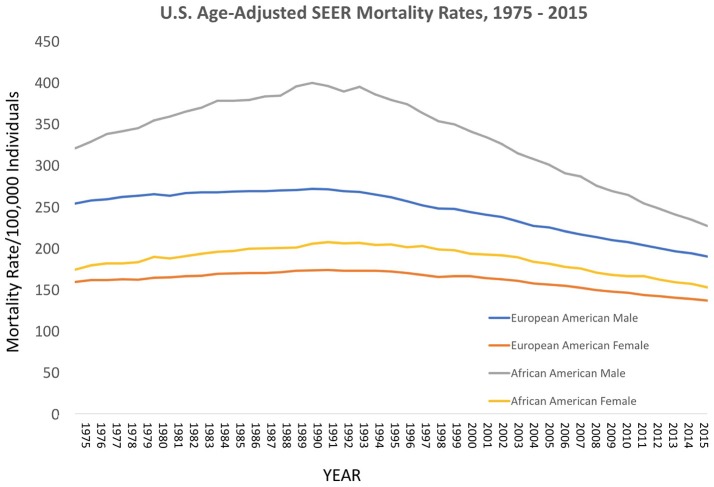
Contemporary cancer mortality by US ethnicity and gender 1975–2013, SEER 2017. https://seer.cancer.gov/report_to_nation/incidence.html (Data Accessed on 08 February 2018).

**Figure 3:**
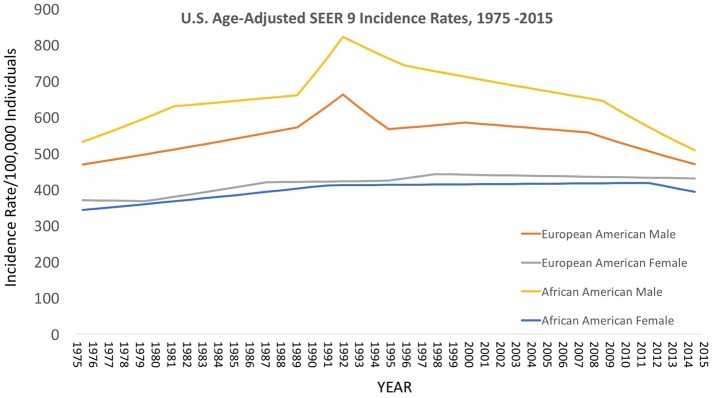
Contemporary cancer mortality by US ethnicity and gender 1975–2013, SEER 2017. (https://seer.cancer.gov/report_to_nation/incidence.html) (Data Accessed on 08 February 2018).

**Figure 4:**
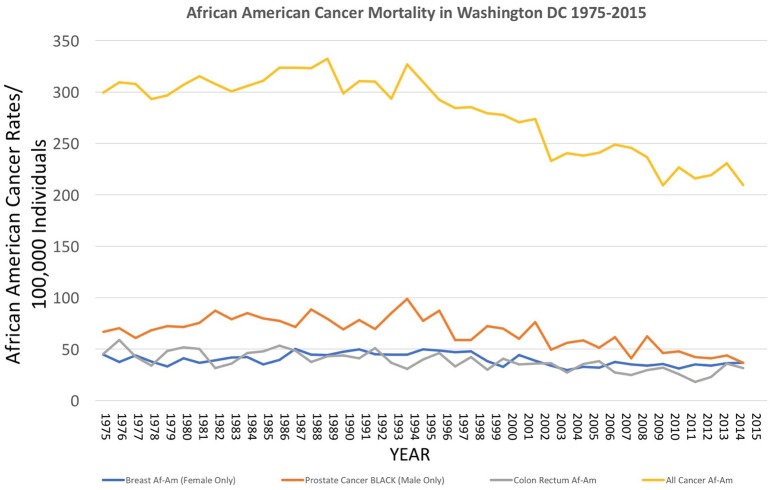
Incidence of cancer mortality, both total and selected types, for African Americans in Washington DC from 1975 to 2015, as reported by the US National Center for Health Statistics (18) https://statecancerprofiles.cancer.gov/historicaltrend/index.php (Data Accessed on 08 February 2018).

Over the decades of the Cobb Collection, the incidence of cancer as a cause of death increased, particularly among AA men. This is consistent with both period and contemporary trends as depicted in Figures 2–4. In both incidence and actual mortality, AA men appear particularly vulnerable to cancer. However, contemporaneously, actual cancer deaths have decreased. The Cobb Collection represents an era when cancer morbidity equaled cancer mortality. The historical pattern of cancer is at variance with contemporary patterns due to the better treatment options now available. Today, cancer can be diagnosed earlier, treated more efficaciously, and people can live longer with many cancers, thus contributing to the current reduction in cancer mortality.

### Geospatial distributions of cancer victims residences

**Figure 5:**
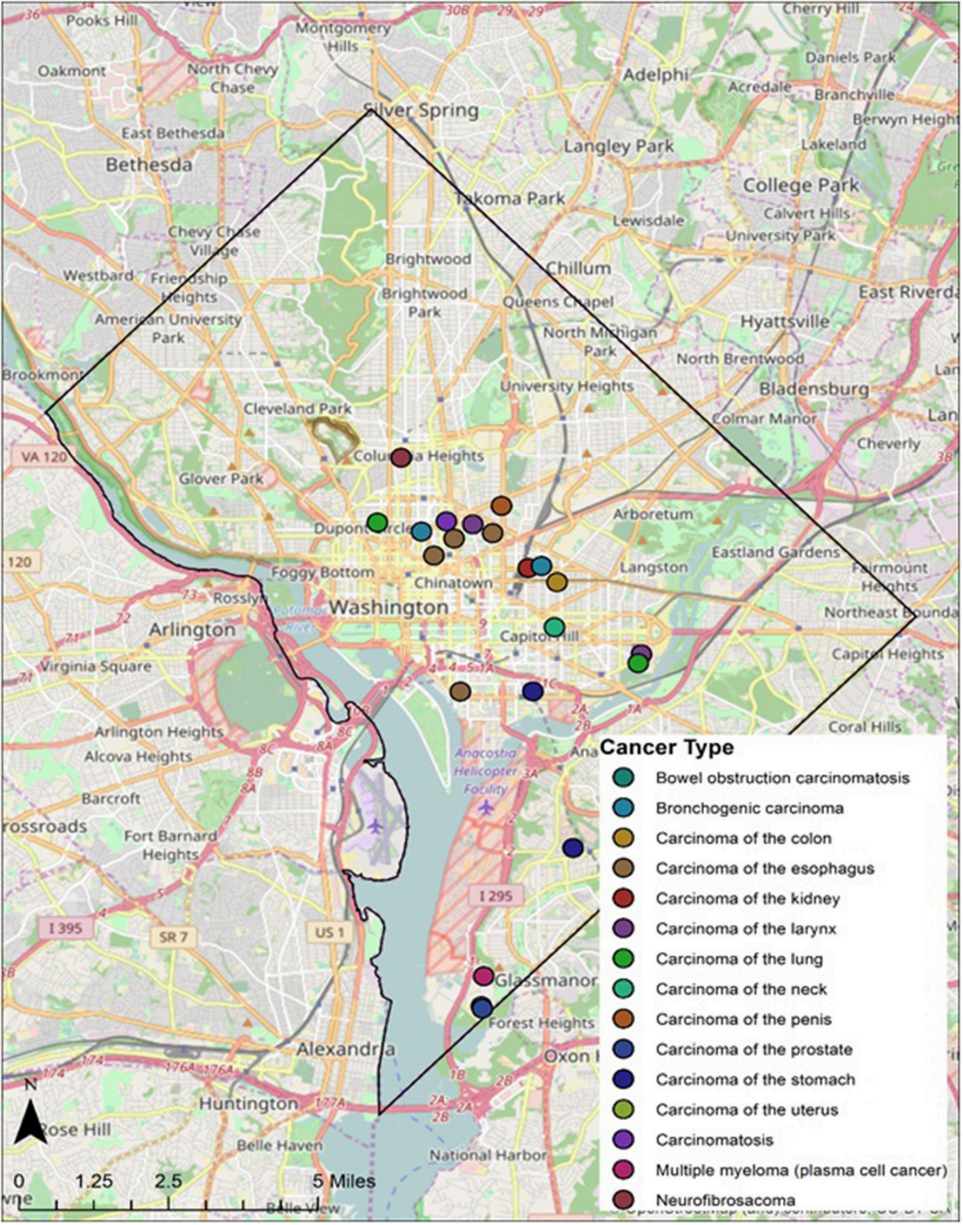
Geospatial distribution of cancer types in Cobb Collection.

**Figure 6:**
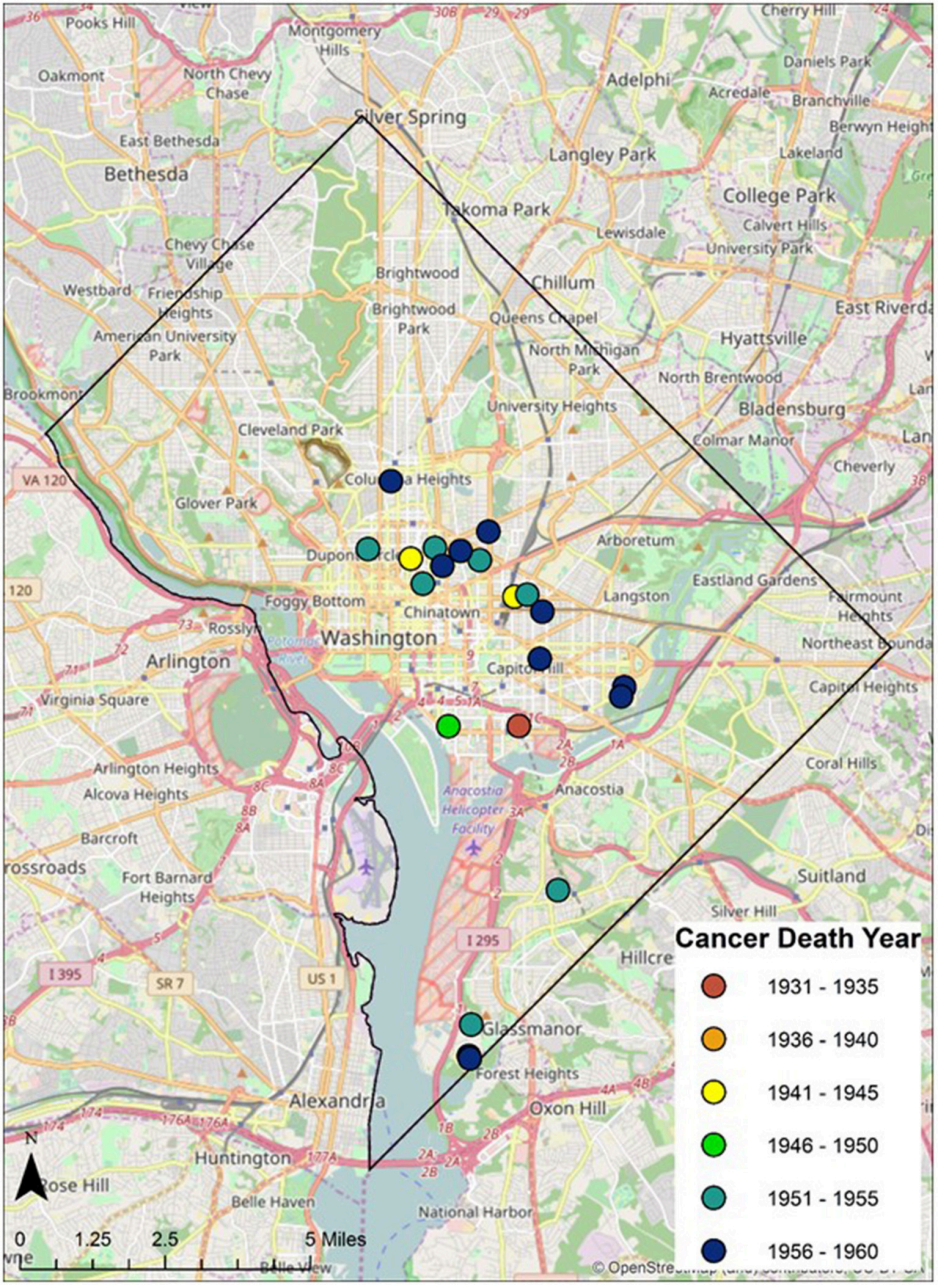
Geospatial distribution of cancer death year in Cobb Collection.

**Figure 7:**
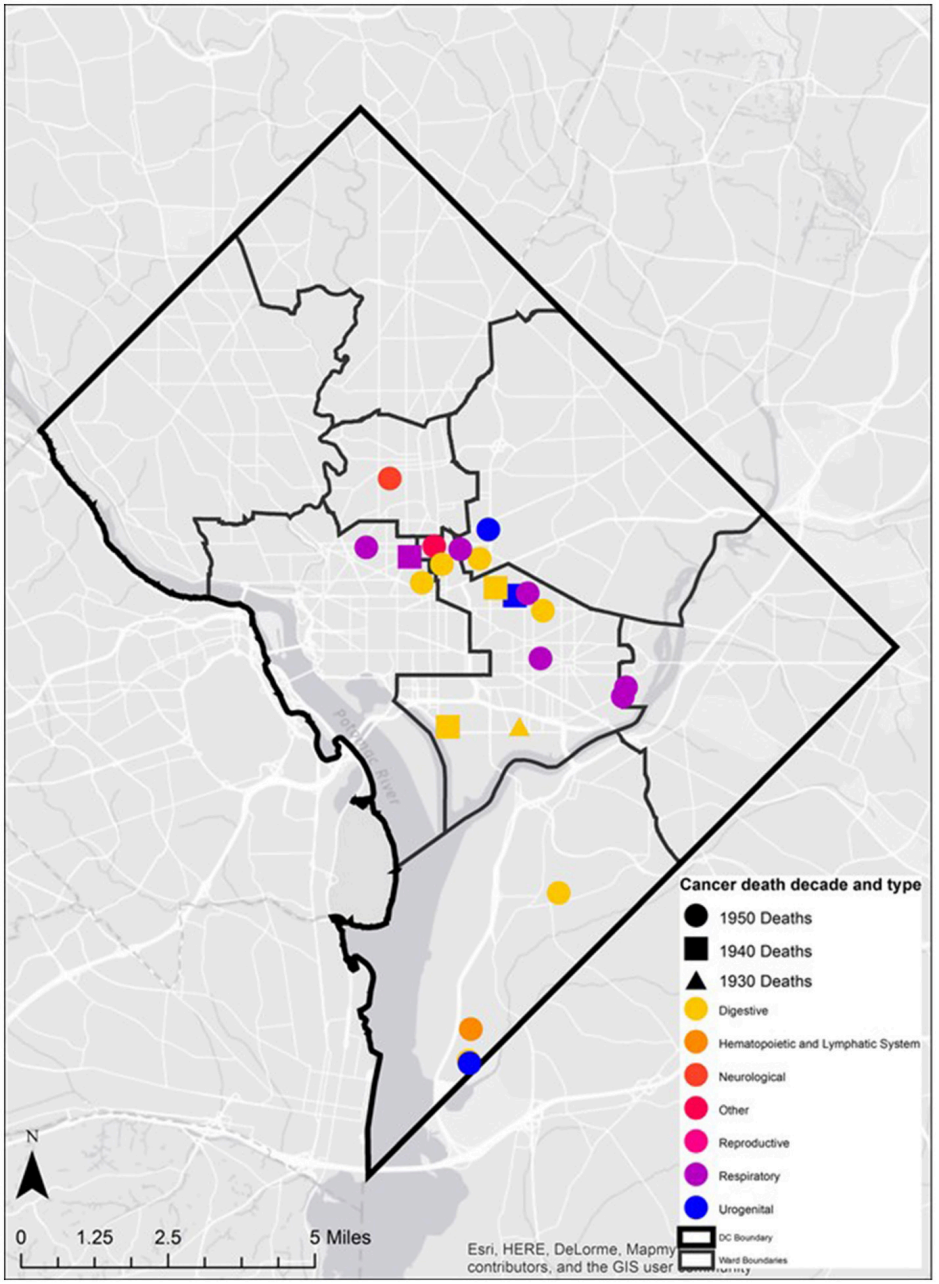
Geospatial distributions of cancer death decade and type in the Cobb Collection.

Twenty-eight individuals were reported to have died from carcinoma as either the primary or secondary cause of death in this historic AA sample. The last known home addresses information revealed 25 of the 28 individuals lived in the District of Columbia. One lived in Virginia and two did not have address information on file. Of the 25 that lived in the city proper, 11 lived in Northwest, five in Northeast, five in Southwest, and four in Southeast. Most individuals were concentrated in central and southern wards with Wards 6 and 8 having the largest counts at nine and five, respectively. Conversely, no individuals resided in Wards 3 and 4. These wards correspond to areas with insignificant black population, when compared to white populations, due in large part to practices of segregation. All other wards had between one and three individuals from the Cobb Collection. All but two of the individuals died in either a nursing home facility or a hospital located in the city. Fifteen died in DC's only public hospital at the time, DC General, located in Southeast. One died in a psychiatric hospital in Southeast. Five died in Northwest hospitals; two in Washington Hospital Center and three at Freedman's Hospital. Five died in a nursing home, three of whom in a facility in Virginia and two in Southwest DC facilities. When the last known residences were mapped geospatial clustering was observed for several of the cancer types (Figures 5–7).

## Discussion

### Cancer types and incidence in the cobb collection

Of the 17 cancer types found in the Cobb Collection, the cancer types with the highest frequencies were bronchogenic carcinoma, carcinoma of the esophagus, carcinoma of the lungs, carcinoma of the larynx and carcinoma of the stomach, collectively accounting for 64% of the total cases of cancer as a cause of morbidity. 68.75% of the cancer incidents with the highest frequencies occurred in AA individuals and 63% in AA males. This is consistent with national trends as AA males consistently bear the greatest cancer mortality over the last several decades than any other group.

The highest frequency cancers are bronchogenic carcinoma and carcinoma of the esophagus, each with four individuals afflicted, all of whom were AA. Bronchogenic carcinoma occurred only in AA males, while esophageal cancer occurred in three AA males and one AA female.

The second most frequent cancer is of the lung, with three male individuals; two AA and one EA. The next highest frequency cancers, each with two individuals, are cancer of the larynx, stomach, and carcinomatosis. All of those afflicted with these cancers were men, with one AA male and one EA male for each. The 11 other cancer types each had one individual diagnosed; five of whom were AA males, three AA females, and three EA males. Deaths due to esophageal and lung carcinoma may have been due to inhalation of cigarette smoke and polluted air. Esophageal cancer cannot be (solely) attributed to alcohol consumption (19) but it may have been a risk factor. Poor oral health coupled with smoking may have also contributed to these mortalities (20).

The results of this study show carcinoma of the esophagus disproportionately affects the AA male, with a 3:1 incidence ratio male to female and 4:0 ratio AA to EA. This may be attributed to environmental factors, which we hope to explore in a follow-up research study. Additionally, increased esophageal cancer rates may also parallel the migration of rural AAs to urban areas in the United States and the downstream ramifications of this transmigration. The AA population increased in Washington DC during the time-frame of the Cobb Collection data from 1931 to 1969. By 1975 African Americans were politically and culturally leading the city with more than 70% of the population (21).

### Age and gender in cancer deaths in the cobb collection

In the Cobb Collection, there are a total of 987 individuals, 780 of which have records of cause of death. Of the 780 individuals, 48% were male and 19% were female and 33% were undetermined. This high proportion of males in the Cobb Collection undoubtedly skewed the specifics of cancer deaths. For example, the Cobb Collection has very low frequencies of female gender-associated cancers.

From the records that are present, there are 26 individuals whose primary cause of death was cancer of some type. There are two individuals with cancer as a secondary cause of death. Of the individuals with records in the Cobb Collection, 6.4% of the males and 2.6% of the females died from cancer.

We note that in our sample, the average age of death by cancer was 65 years. Over time, the lifetime incidence of cancer tends to increase as individuals age. Cancer is more commonly seen in older individuals due to their greater accumulated exposure to carcinogens and increased probability of developing deleterious mutations affecting cell division. Upon entering midlife, many individuals who ultimately die from cancer have been exposed to multiple cancer risk factors and thus express elevated incidence rates for different cancers (22). The fact that the Cobb Collection is disproportionately represented by older individuals and men likely influences both the specific kinds of cancer present and the overall rates of cancer, and this must be taken into consideration in retrospective analyses of such historical samples. The greater incidence of cancer among the older individuals in the Cobb Collection could also be attributed to their proportionately lower incomes for age. Younger and more educated individuals could use a smaller portion of their incomes toward healthcare whereas older individuals with limited access to important socioeconomic factors such as clean housing, adequate diets, access to appropriate and adequate healthcare, and available care givers at home may have increased risk for cancer becoming a cause of death.

Although improved diagnosis of cancer probably accounts for some of the increase in recognized cancer mortality in the Cobb Collection, there is also likely underrepresentation of cancer in the Cobb Collection, as our observed results are less than would have predicted from national norms It is important to mention social stigma and myths associated to cancer may have played a role in underrepresented diagnosis as people may not have sought care when sick, agreed to treatment if diagnosed, and may have developed other co-morbidities (e.g., metabolic syndrome) that may have been recorded as the primary cause of death as well as predisposed some the Cobb Collection individuals to cancer. In 1961, it was reported that nearly 90% of US doctors did not disclose cancer diagnoses to their patients as they believed it could cause them harm. The advent of psycho-oncology in the 1970s improved doctor-patient communication about cancer (23).

### Comparison of cobb collection cancer with international classification of diseases for oncology

Since 1976, the International Classification of Diseases for Oncology (ICD-O) has been used in healthcare for coding the topography and histology of neoplasms. Its history begins with the early formation of the International Statistical Classification of Diseases (ICD), created in 1893 and overseen by the World Health Organization (WHO) after World War 2 and the establishment of the United Nations. The first few issues of the ICD simply classified the existence of a neoplasm, and later would specify the region. With the publication of the Sixth Revision of ICD in 1948, the classification of neoplasms was still based primarily on topographic site but also listed behavior, noting whether the neoplasm is malignant, benign, or not specified. Except for lymphatic and hematopoietic neoplasms, choriocarcinoma, melanoma, and certain benign neoplasms, there had been no coded nomenclature for other histologic types (16) http://apps.who.int/iris/bitstream/10665/96612/1/9789241548496_eng.pdf (24).

The American Cancer Society (ACS) published specific coding for neoplasms tin 1951 as the *Manual of Tumor Nomenclature and Coding* (MOTNAC), and saw widespread use across the world in the ensuing years. A new edition of MOTNAC appeared in 1968 (a year before the last cadaver was added to the Cobb Collection but a year after the last individual dying of cancer was recorded), but oncologists were realizing that knowledge solely of the site or topography of a tumor was not sufficient for planning treatment or conducting research. Incidence and survival rates differed according to the histologic type of the tumor.

To this end, WHO published the first edition of the ICD-O in 1976, which had a topography section based on the malignant neoplasm rubrics of ICD-9 and a morphology section that was a one-digit expansion of the MOTNAC nomenclature. The second edition ICD-O, published by WHO in 1990, and the third edition of ICD-O, published in 2000 with a revision in 2011, further refined the classifications of neoplasms, optimizing for use in cancer registries, pathology departments, and research departments specializing in Sudhakar (24) and WHO (16).

When the cancer types seen in the Cobb Collection are compared with the ICD listings, we observed that over time, there is an increase in the diversity and numbers of cancers identified as causes of death in Cobb Collection individuals. Over time there is also increased variability in the affected body regions. This may reflect increased sophistication in the clinical diagnosis of cancer as well as increased specificity in the official cancer categories.

### Geospatial environmental influences on historic cancer

There were some limitations in researching environmental toxicant exposures in historic DC in the context of this paper. Since the Environmental Protection Agency was not founded until 1970, scientific studies on the magnitude and impact of chemical contaminants were not abundant during the decades of interest for this study (1930–1969). Additionally, carcinogenic compounds may have been present in air pollution and water contamination during earlier decades before EPA regulations on chemicals and toxic emissions were restricted or banned.

Since 1955, the Public Health Service has researched air pollution and revealed in their studies that an estimated 308 urban places in the United States with a population of 2,500 or more have a “major” air pollution problem. The greater Washington DC area, comprising parts of northern Virginia, Maryland, and West Virginia, is defined as a Standard Metropolitan Statistical Area (SMSA). In this 1963 report for the Public Health Service, the extent of air pollution increases as an SMSA population reaches 1,000,000 or more. Urban centers are also more likely to have severe problems in air pollution. In 1950, there were about 44.5 million people living in these high density areas. By 1960, this number increased to 62.8 million in 22 areas in the United States (26).

To examine the extent of chemical contaminants in the DC environment, a study in 2011 investigated sediment cores in the Anacostia River watershed. These cores provide clues to determine chronology of pollutants in the airshed, watershed, and ground water systems. The Anacostia River cuts through the city, past the Capitol, and runs into Montgomery County and Prince George's County, Maryland. According to the EPA, the Anacostia watershed is among the most urbanized in the country and has lost over 70% of its forestland and 6,500 acres of wetland.

The Potomac River Basin comprises all of Washington, DC, including the Anacostia watershed, as well as parts of northern Virginia, eastern West Virginia, central and western Maryland, and southern Pennsylvania. In 1993, a study by the US Geological Survey investigated numerous types of pesticide run off in the Potomac River Basin surface water, bottom material, ground water, and fish tissue. The great majority of pesticide use in the area was from agriculture, which included use of atrazine and paraquat (27). Atrazine and paraquat, among other pesticides, have been linked to carcinogenesis (28). In addition to atrazine and paraquat, DDT and HCH were also used in the 1950s and 1960s for agriculture and mosquito control operations. Surveys conducted by the US Geological Survey revealed residues of these chemicals in the Potomac River basin and adjacent areas. The study found that pesticides were along the main stream of the river and included a number of recently banned insecticides that were in use in the Potomac River Basin for some time. Overall, it was found that pesticides appear to be in high concentrations in fish tissue than in water and bottom material, with fish tissue as good indicators of contaminant levels (27). It is worth noting that for many of the AA migrants to DC with origins in the rural South, fishing in the Potomac River and its tributaries would have been an accepted means of augmenting their diets. In addition these pathways of exposure, workplace exposure of people to chemicals was greater in 1930s through 1950s before OSHA regulations were enforced for chemical emissions in occupational settings (e.g., manufacturing factories).

According to the American Cancer Society, alcohol consumption is a risk factors for cancers of the mouth, pharynx, larynx, esophagus, stomach, liver, colorectum, and female breast. Heavy alcohol drinking paired with tobacco smoking significantly increases one's risk of developing esophageal, mouth, and larynx cancers than either smoking or drinking alone (29). The results of this study show carcinoma of the esophagus disproportionately affects the AA male, with a 3:1 incidence ratio male to female and 4:0 ratio AA to EA. The geospatial locations of carcinoma of the esophagus were clustered in the Ward 6 section of downtown DC (Figure 5). This pattern likely reflected the prevailing discriminatory residential housing prevalent in DC until the 1970s with AAs clustered in a few of the city's wards.

Increased esophageal cancer rates also parallel the migration of AAs to urban areas in the United States. The population of AAs in DC increased significantly in DC in the 1930s and steadily rose until 1990, when the city began to see effects of gentrification reversing the trend of prior decades. According to US Census, the AA population in the nation's capital peaked in 1970 to 71.1%. A decade later, in 1981, it was reported that the DC revenue for alcohol sales far exceeded national average, with liquor sale (and presumably consumption) four-times the average and wine three-times the national average (30).

Low socioeconomic status is a risk factor for smoking and heavy drinking, in addition to lack of exercise and limited access to health care facilities. In DC, there are five hospitals in Northwest, one in Northeast, and one located in Southeast that serves about 20% of the district's total population. All cancer centers in DC are all located in the Northwest quadrant. The further one lives away from one of these centers the greater the negative impact it could have on their access to care and level of compliance with treatment options. In regards to the historic consideration of this study, it is presumed that the individuals who died of cancer were likely diagnosed some time before their death. If one lived in close proximity to a hospital in the city that provided oncology treatment, then that individual would likely have better access to care and better chance to survive than someone who lived much further away.

From the results of the data in this study, AA males were the only patients to have bronchogenic carcinomas. This suggests the AA males living in Washington, DC during the examined decades had a substantial risk in developing lung and bronchus malignancies, with increased incidence over time. To adequately analyze this, it must first be considered the disparities that exist for the AA male, and, second, what factors influence a trend of increased incidence over time.

Lung and bronchus cancer is the leading cause of cancer deaths in the US (29) with an increased burden to AA men and low-income Americans in general. Given that cigarette smoking is the leading cause of lung cancer in this country, it is significant to investigate disparities in smoking tobacco. Cigarette smoking peaked in the United States from 1940 to 1970, with a steady decline in incidence since 1980. AAs and Hispanics are actually less likely to smoke in adolescence as compared to EAs, but are more likely to begin smoking in adulthood and less likely to quit than EAs. This places AAs and Hispanics at considerable risk for subsequently developing lung cancers, with AAs more likely to die from these cancers. In 2009, AAs were 66% less likely to receive timely treatment as compared to EAs. Additionally, lung cancer afflicts more males than females in this country, and is more prevalent in AA males than EA males as recorded trends from 1935 to 1969 suggest. The increased incidence of lung cancer in AA males could be attributed to increased access to medical facilities over time, and to increased exposures to risk factors resulting from migration to urban areas (31). This supports the trend observed in our results that AA males are most likely to die from cancer. When placed in the context of DC demography changes, there is a striking parallel of increased AA immigration into the city over time, including the decades investigated in this study. By 1990, there has been a steady decline of the district's black population as a result of gentrification.

Data in 2010 showed that the burden of cancer affects AAs and low-income Americans to a much larger degree than among other groups. The incidence of lung and bronchus carcinomas is greatest in DC's wards 5 and 7, with mortality rates highest in wards 4, 5, and 8. Historically, wards 7 and 8 are the poorest in the city and the most abundant in AA populations.

A study published in 1972 examined incidence of malignant tumors diagnosed in “Negroes” at 11 major hospitals in DC from 1965 to 1969. There was a total of 6,228 cases of carcinoma with 3,163 in males and 3,065 in females. The most common cancer types in AA males were prostate, bronchogenic, and esophageal. This matches data from the Cobb Collection in that bronchogenic and esophageal cancers predominated in this same demographic group. The low prevalence of prostate cancer in the Cobb Collection is interesting, however. The most common cancer types in AA females in this 1972 report were breast, cervix uteri, and colon (32). In contrast to our data, this 1972 study found that between the ages of 20 and 49, the age-specific incidence of carcinomas were higher in Negro females than Negro males. The study attributes this gap to high rates of breast and cervical cancers, although the incidence rate of breast cancer was higher in EAs, even at that time. Given the fact we have only four females in our study, all of whom are AA, the comparison to this study in regards to the burden of cancer on females may be limited by low statistical power. There was one case each of cervical, uterine, esophagus, and bowel obstruction carcinomatosis in the four females. It should be noted that bowel obstruction carcinomatosis is often a complication from abdominal, ovarian, or colon cancers. Interestingly, we did not have a single incidence of breast cancer in the Cobb Collection. This could suggest that females may have been diagnosed with cancer, but survived it more often than males who were diagnosed and died of cancer. Additionally, because rates were higher in EA females, there may not have been enough cases to match the trend seen in the late 1960s.

Kovi and Heshmat compared the Washington, DC data to AAs and EAs in Alameda County, California and to that of Africans in Ibadan (Nigeria), Kyadondo (Uganda), and Natal (South Africa). The study supported the view that the overall incidence of cancer was much higher in both AAs and EAs compared to Africans. Esophageal carcinoma in AA males was reported as high in Washington, DC, whereas their EA counterparts in Alameda, California showed low rates. A study in 1970 reviewed patterns of esophageal cancer in the United States from 1930 to 1937, which showed a rapid increase in esophageal malignancies among the non-EA populations, while EAs had no substantial change in incidence rates during those decades. This was suggested to be associated with an increased cigarette smoking and alcohol consumption pattern that was more pronounced in urban settings. It was also surmised that heavy drinking was more common in AAs than EAs of similar socioeconomic class (32, 33). Additionally, rates of bronchus carcinomas were very high in AA males, and intermediate in AA females and EAs in California (32). This parallels our data as AA males had much higher incidence of lung and bronchogenic carcinoma as compared to EA males by a 6:1 ratio.

A study in 1993 sought to explain the racial/ethnic disparity of higher rates of oral cancers in AAs compared to EAs. AA men had higher incidence of oral cancer than EA men in the 1960s, but by the mid-1980s, AA men were twice as likely to die of oral cancer as EA men. The study found that there were higher proportion of heavy drinking among AAs compared to EAs and that when this was combined with heavy tobacco smoking, AAs were more likely to develop oral cancer than EAs by a ratio of nearly 200:40. The study suggests that heavy drinking and smoking risk factors combined in an individual multiplied the enhanced risk for development of cancer (34).

### Relevance of historic cancer to contemporary health disparities

The research we report on historic cancer in Washington DC AA is important because these data enrich our understanding of the historical medical template upon which contemporary health disparities in cancer are perched. More knowledge of this historic health template can produce a better appreciation of contemporary medical statistics and assist healthcare professionals in the sustainable remediation of current cancer health disparities.

Although cancer mortality has declined in recent times (35), cancer is still one of the leading causes of death among AA. With the cancer morbidity rates among AAs on the rise, the need for adequate preventative methods and care is exceedingly important. This study provides a starting point in this area, as it examines the trends in over four decades within a subgroup of recognized vulnerability. We have analyzed the trends in this historic AA group by age, gender, cancer types, and affected body region to develop a heretofore unknown profile of cancer morbidity. This research lays a foundation for future studies that can suggest preventive strategies such as dietary management, smoking cessation, and improvements to the inner city environment. These integrative approaches should embrace the “public health exposome” as an approach that would allow researchers to encompass spatio-temporal analyses of the effects of the natural-, built-, and social environments on health outcomes (36, 37). Given our findings on the historic Washington DC African Americans of the Cobb Collection, we see this strategy to comprehensively addressing current health disparities.

The Cobb Collection's cancer subgroup can act as a localized historical reference point for contemporary investigations of cancer health disparities in the District of Columbia, among AA, and, by extension, to the larger American population. Our identification of the historic incidence of specific cancers among AA of Washington DC may help researchers calculate the persistence of particular types of cancer, the depths of the legacy of ethnic disparities, and potentially develop hypotheses about local prevention methods specific to the peoples of the District of Columbia.

## Author contributions

LJ provided bioinformatic and computational assessments of the biological data. HJ contributed geographical science evaluations of the residential patterns for cancer patients. MM assisted with the historical bibliographic contexts for the research. NG provided clinical insights into historic cancer. SK contributed epidemiological perspectives on cancer in diverse populations. RO did the initial studies on cancer patients in the Cobb Collection. FJ conceived of the project, organized the research team, and integrated the various sources of data.

### Conflict of interest statement

The authors declare that the research was conducted in the absence of any commercial or financial relationships that could be construed as a potential conflict of interest. The reviewers AR and MAT and the handling Editor declared their shared affiliation.

## Footnotes

^1^National Cancer Institute. *Cancer Types*. Available online at: https://www.cancer.gov/types (Accessed June 09, 2017).
